# Supplementary material for: Order-disorder charge density wave instability in the kagome metal (Cs,Rb)V3Sb5
Source: Nat Commun. 2023 Feb 23;14:1015. doi: 10.1038/s41467-023-36668-w (PMC9950456; doi:10.1038/s41467-023-36668-w)
Supplement: Supplementary file 1 — Supplementary Information [file 41467_2023_36668_MOESM1_ESM.pdf]

Supplementary Information for

Order-disorder charge density wave instability in the kagome metal (Cs,Rb)V_3Sb_5

D. Subires[1], A. Korshunov[2], A. H. Said[3], L. Sánchez[1], Brenden R. Ortiz[4], Stephen D. Wilson[4], A. Bosak[2], and S. Blanco-Canosa[1,5*]

[1]Donostia International Physics Center (DIPC), San Sebastián, Spain
[2]European Synchrotron Radiation Facility (ESRF), BP 220, F-38043 Grenoble Cedex, France
[3]Advanced Photon Source, Argonne National Laboratory, Lemont, IL 60439
[4]Materials Department and California Nanosystems Institute, University of California Santa Barbara, Santa Barbara, California, 93106, USA.
[5]IKERBASQUE, Basque Foundation for Science, 48013 Bilbao, Spain

* sblanco@dipc.org

1) Diffuse scattering (DS) of RbV_3Sb_5.

We have also performed diffuse scattering on the RbV_3Sb_5 single crystals, observing the same diffuse profile as the CsV_3Sb_5 samples. The DS measurements were performed at the ID28 station at ESRF [1].

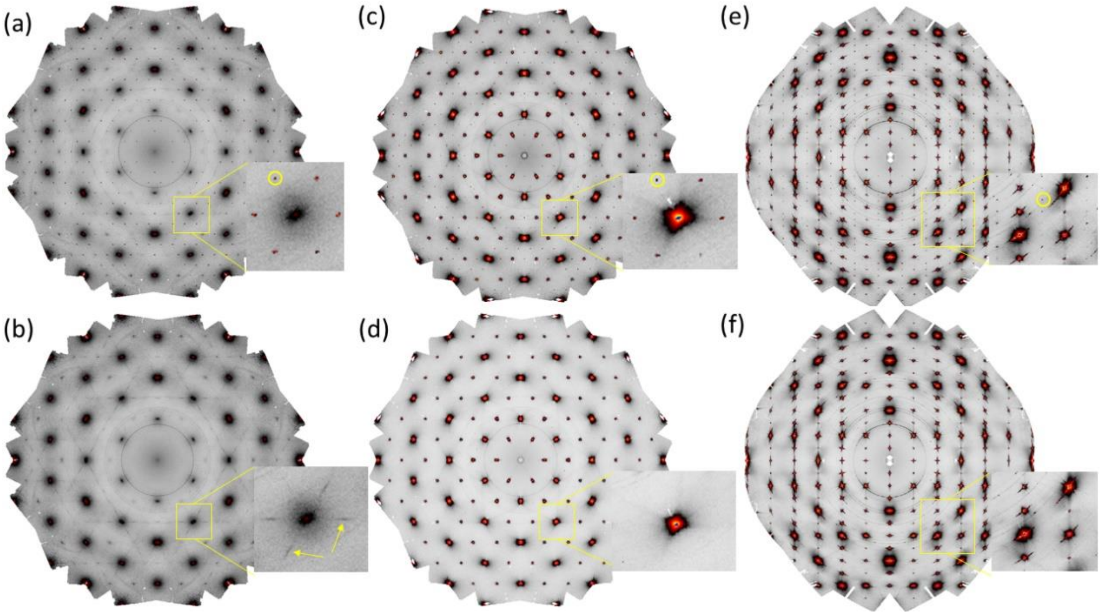

Supplementary Figure 1. Diffuse scattering at 100 K and 110 K for RbV_3Sb_5 for the (a) (*h k* 0.5) plane, showing the CDW Bragg reflections (weak spots highlighted within the yellow circle in the zoomed in area) and the precursor of the 3D CDW, marked with arrows. (b) and (c) (*h k* 0) and (*h* 0 *l*) planes, respectively (no precursor).

2) Phonon dispersion and mode assignment in CsV_3Sb_5.

Figure 2 zooms in into the low energy phonon branches around the *L* point. The calculation was carried out by means of Density Functional Perturbation Theory

(DFPT) as explained in the main text using Grimme's semiempirical approach for the van der Waals corrections [2-4].

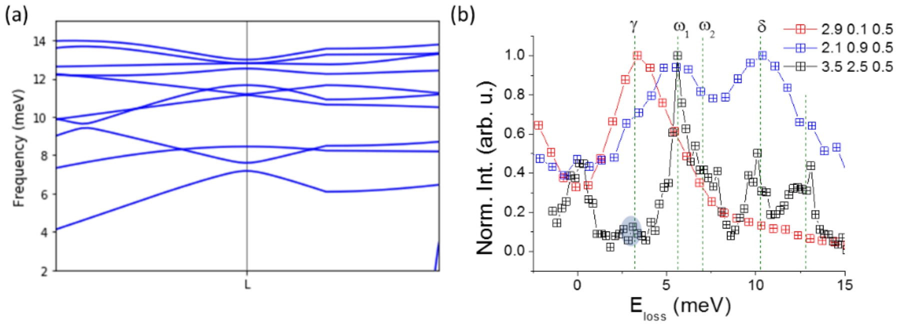

Supplementary Figure 2: (a) Zoom-in of the L point phonon dispersion of CsV_3Sb_5 calculated by Density Functional Perturbation Theory. (b) Identification of the phonon modes.

3) <u>Phonon fitting procedure</u>

Here we describe the phonon fitting procedure of figure 4(e). In the range of 12 meV there are 6 modes at the L point (see DFPT calculations in suppl. Inf. Figure 2(a)), 4 of them visible in the IXS spectra and labelled as δ, ω_1, ω_2 and δ. The two phonons ω_1 and ω_2 are very close in energy and overlap, thus a reliable fitting that reflects the experimental trend of the linewidth turns out to be a challenge.

The fitting procedure was carried out following the steps:

- First, we fit the IXS spectrum at 105 K and obtain the ω_1 and ω_2 linewidths. From this, we see that ω_1 is resolution limited.
- Looking at the raw data, both ω_1 and ω_2 increase their linewidth at 96 K and at higher temperatures as compared with 105 K scan. Trying to fit the linewidth of ω_1 and ω_2 '*from scratch*' (namely, allowing the code to reach convergence by itself) gives random values in both ω_1 and ω_2 that do not reflect the experimental trend, a T-dependent broadening.
- Therefore, we opted for fixing the linewidth of ω_2 to the value obtained at 105 K and fit ω_1 for the rest of the temperatures. The raw data shows that ω_2 broadens less than ω_1, or its broadening is merely an effect of the larger broadening experienced by ω_1. By fixing the linewidth of ω_2 during the whole fitting process, its error is zero, and this also gives an anomalously low error for ω_1.
- To circumvent this issue, now we have continued with the fitting procedure by releasing the linewidth of ω_2, thus allowing an uncertainty in ω_2, and continue with the fitting until convergence is reached.

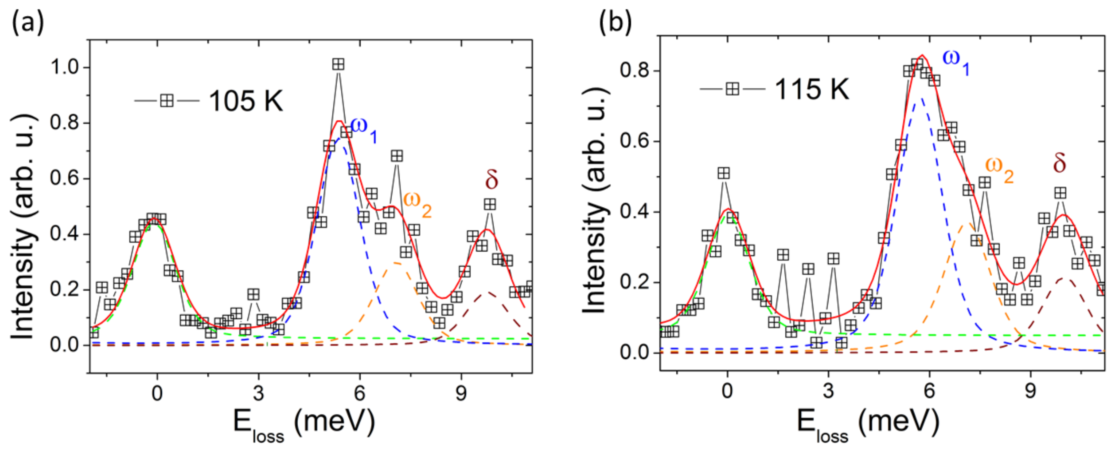

Supplementary Figure 3: Fitting of the (a) 105 K and (b) 115 K.

We want to point out that the temperature dependence of ω_1 we show in the main text reflects a linewidth trend, as observed experimentally, and we warn the reader not to be interpreted it as the intrinsic phonon lifetime of ω_1.

References

[1] A. Girard et al., A new diffractometer for diffuse scattering studies on the ID28 beamline at the ESRF. Journal of Synchrotron Radiation, 26, 272 (2019).

[2] P. Giannozzi, Advanced capabilities for materials modelling with QUANTUM ESPRESSO. Journal of Physics: Condensed Matter .29, 465901 (2017).

[3] S. Grimme, Semiempirical GGA-type density functional constructed with a long-range dispersion correction. Journal of Computational Chemistry 27,1787-1799 (2006).

[4] J. Diego et al., van der Waals driven anharmonic melting of the 3D in VSe_2. Nature Communications 12, 598 (2021).
